# Expression of hypothalamic-pituitary-gonadal axis-related hormone receptors in low-grade serous ovarian cancer (LGSC)

**DOI:** 10.1186/s13048-016-0300-5

**Published:** 2017-01-25

**Authors:** Zheng Feng, Hao Wen, Xingzhu Ju, Rui Bi, Xiaojun Chen, Wentao Yang, Xiaohua Wu

**Affiliations:** 1 Department of Gynecological Oncology, Fudan University Shanghai Cancer Center, 270 Dong-an Road, Shanghai, 200032 China; 2 Department of Oncology, Shanghai Medical College, Fudan University, Shanghai, 200032 China; 3 Department of Pathology, Fudan University Shanghai Cancer Center, Shanghai, 200032 China

**Keywords:** Low-grade serous ovarian cancer, Hormone receptor, Prognosis

## Abstract

**Background:**

The aim of our study was to investigate the clinical features and expression levels of hypothalamic-pituitary-gonadal axis-related hormone receptors in low-grade serous ovarian cancer (LGSC).

**Methods:**

We retrospectively investigated the clinical features of 26 consecutive patients with LGSC who underwent primary staging or debulking surgery between April 2005 and June 2013 in our center; concomitant primary high-grade serous ovarian cancer (HGSC) patients were randomly selected at a 2:1 ratio for comparison. Tissue microarrays were constructed from the LGSC and HGSC specimens, and the expression levels of six hormone receptors in the hypothalamic pituitary-gonadal axis were analyzed by immunohistochemistry.

**Results:**

The median (range) age of patients with LGSC was 54 (27–77) years. According to the FIGO staging system, the cases were distributed as follows: stage I, 6 (23.1%); stage II, 0 (0%); stage III, 19 (73.1%); and stage IV, 1 (3.8%). The 2-year and 5-year overall survival rates for LGSC were 91.8% and 67.5%, respectively. The expression levels of the hormone receptors were as follows: ER, 80.8%; PR, 34.6%; AR, 53.8%; FSHR, 84.0%; LHR, 65.4%; and GnRHR, 100%. Hormone receptor-positive patients had a better prognosis compared with hormone receptor-negative patients, but the difference was not significant.

**Conclusions:**

Our study presented a higher overall survival rate and distinctive hormone receptor expression levels of LGSC patients compared with the HGSC cohort. Patients with positive hormone receptor expression tended to have a better prognosis than the corresponding hormone receptor negative patients.

**Electronic supplementary material:**

The online version of this article (doi:10.1186/s13048-016-0300-5) contains supplementary material, which is available to authorized users.

## Background

Ovarian cancer is one of the most common and lethal malignancies among females worldwide. The standard treatment for ovarian cancer includes staging/debulking surgery and individual platinum-based adjuvant chemotherapy, regardless of the tumor’s histologic subtype [1, 2]. However, previous studies have shown that epithelial ovarian cancer is not a single disease but rather a group of heterogeneous tumors based on distinct morphologic and molecular genetic features [3]. The “one-size-fits-all-concept” has been challenged for the treatment of some rare ovarian cancer subtypes [4–6].

Low-grade serous ovarian cancer (LGSC) represents less than 10% of serous ovarian cancers and is less sensitive to conventional platinum-based chemotherapy compared with high-grade serous ovarian cancers (HGSC) [5–12]. Previous studies have shown considerable estrogen receptor (ER) and progesterone receptor (PR) expression levels in LGSC [13, 14], and hormone therapy might be a promising alternative for recurrent LGSC [15]. However, the expression levels of other hormone receptors related to the hypothalamic-pituitary-gonadal axis (androgen receptor (AR), follicle-stimulating hormone receptor (FSHR), luteinizing hormone receptor (LHR) and gonadotropin-releasing receptor (GnRHR)) in LGSC, which could also mediate the effects of steroid hormones on the development and progression of ovarian cancer, have not been shown. In addition, due to the rarity of LGSC, studies on the clinicopathologic characteristics of this type of ovarian cancer are quite limited.

The aims of this study were twofold: (1) to assess the expression levels of hormone receptors related to the hypothalamic-pituitary-gonadal axis and their prognostic significance in LGSC and (2) to further delineate the clinical features and prognosis of LGSC patients in comparison with concomitant HGSC patients at a single institution.

## Methods

### Clinical data

This study was conducted according to the Declaration of Helsinki and was approved by the Committee at Fudan University Shanghai Cancer Center. Written informed consent was obtained from all individual participants included in the study.

We retrospectively investigated women who underwent primary staging or debulking surgery for LGSC at Fudan University Shanghai Cancer Center with available paraffin specimens between April 2005 and June 2013. Patients were excluded if they had received neoadjuvant therapy prior to the primary surgery, or were found to have other histological diagnoses upon pathological review. Concomitant primary HGSC patients were also investigated for comparison.

Clinical and pathological data were obtained from medical records, cancer registries, and pathology reports. Patient characteristics, including age, FIGO stage (according to the 2005 system), surgical outcomes, date of primary surgery, date of progression or recurrence, date of last follow-up, and the patient’s disease status at last contact were collected. Twenty-six consecutive LGSC patients and 875 concomitant HGSC patients were identified, and we use SPSS software for simple random sampling to select 52 HGSC patients at a 2:1 ratio for comparison. All of the patients were followed up with until December 31^st^, 2014.

R0 was defined as the absence of macroscopic residual disease (RD) after surgery. Chemosensitivity was defined as a time interval of 6 months or longer between the completion of platinum-based chemotherapy and the detection of relapse. Chemoresistance was defined as disease progression during adjuvant platinum-based chemotherapy or within the 6-month interval between the completion of chemotherapy and the detection of relapse. Overall survival (OS) was defined as the time interval from the date of the primary surgery to the date of death or the last follow-up.

### Tissue microarray and immunohistochemistry

The histological diagnoses were based on the WHO criteria [16], and all microscopic slides were reviewed by two experienced gynecologic pathologists. A microarray (1 mm) using triplicate tissue samples from each tumor was prepared [17, 18]. Sections (3 μm) of the completed tissue microarray were analyzed by standard immunohistochemistry protocols. Immunohistochemical staining was performed in all cases for ER and PR using a Ventana Benchmark XT autostainer (Ventana Medical Systems Inc., Tucson, AZ, USA). Staining for AR, FSHR, LHR and GnRHR was performed using the Envision horseradish peroxidase system following the manufacturer’s protocol (DAKO EnVision System K5007). The details of the primary antibodies used in this study were as follows: ER (Roche SP1), PR (Roche 1E2), AR (Abcam ab133273, 1:100), FSH-R (Abcam ab150557, 1:100), LH-R (Santa Cruz sc-25828, 1:40), and GnRH-R (Abcam ab183079, 1:50). Negative (no primary antibody) and positive (according to the primary antibody instructions) controls were included in each staining experiment.

Results were independently judged, evaluated, and scored by two experienced gynecologic pathologists that were blinded to patient information. The results were judged as the numerical mean of the values obtained from the triplicate scores. The expression of hormone receptors was determined using the following criteria:

ER, PR, and AR levels, >10% showing positive nuclear staining of any intensity were defined as positive [19, 20].

FSHR and LHR levels, evaluation of the cytoplasmic staining reaction was performed in accordance with the immunoreactive score (IRS). The IRS was defined as the staining intensity (SI) multiplied by the percentage of positive cells (PP). SI was defined as 0 (negative), 1 (weak), 2 (moderate) and 3 (strong). PP was defined as 0 (negative), 1 (no more than 10% positive cells), 2 (11% to 50% positive cells), 3 (51% to 80% positive cells) and 4 (greater than 80% positive cells). IRS = SI × PP, IRS ≥ 3 was defined as positive [21].

The cytoplasmic staining of GnRHR was recorded as negative, weak, moderate and strong. Staining of any intensity was regarded as positive [22].

### Statistical analyses

SPSS software (version 21.0) and GraphPad Prism (version 6.0) were used for the statistical analyses. Descriptive statistics were used for the demographic data and were summarized as the medians with ranges or the frequencies with percentages. The categorical data were compared with chi-squared or Fisher’s exact tests as appropriate. OS were analyzed with the Kaplan-Meier method, log-rank tests and cox regression analyses for univariate analyses. No significant impact of clinicopathologic parameters on OS was observed in the univariate analyses, so multivariate analysis was not performed. *P* < 0.05 was considered statistically significant, and all reported *p*-values were 2-sided.

## Results

### Patient clinical features

A total of 901 serous ovarian cancer patients received their primary surgery at our institution. Twenty-six (2.9%) of these cases were LGSC and 875 (97.1%) were HGSC. Among them, 52 primary HGSC patients were randomly selected at a 2:1 ratio for comparison. The clinical features of LGSC cases are listed in Table 1. The clinicopathologic features of the LGSC and HGSC cohorts are compared in Table 2.

**Table 1:** Clinical features of LGSC cases

Case no	Age	FIGO stage	Residual disease	Chemotherapy	Chemosensitivity	Follow-up (months)	Survival Outcomes
1	57	IIIC	R0	Y	Y	21	Censored
2	39	IIIC	≥1 cm	Y	Y	20	Alive
3	77	IC	R0	NA	NA	NA	Censored
4	27	IIIC	≥1 cm	Y	N	18	Died
5	46	IIIC	≥1 cm	Y	N	32	Alive
6	68	IIIC	0.1-1 cm	Y	NA	12	Died
7	49	IIIC	0.1-1 cm	Y	N	52	Alive
8	42	IIIC	≥1 cm	Y	N	13	Censored
9	76	IIIB	R0	Y	Y	22	Alive
10	57	IIIC	0.1-1 cm	Y	Y	20	Alive
11	54	IIIC	0.1-1 cm	Y	N	19	Alive
12	75	IIIC	R0	Y	N	70	Alive
13	61	IB	R0	Y	NA	19	Censored
14	39	IB	R0	Y	Y	56	Alive
15	54	IIIB	0.1-1 cm	Y	Y	53	Alive
16	34	IC	R0	Y	Y	30	Alive
17	60	IIIC	R0	Y	Y	41	Alive
18	40	IA	R0	Y	Y	20	Alive
19	40	IA	R0	Y	Y	59	Alive
20	53	IIIC	0.1-1 cm	Y	N	30	Died
21	57	IIIC	R0	Y	Y	22	Alive
22	43	IIIC	0.1-1 cm	Y	Y	45	Alive
23	35	IIIC	R0	Y	Y	87	Alive
24	55	IIIC	R0	Y	Y	50	Alive
25	69	IV	0.1-1 cm	Y	Y	27	Died
26	62	IIIC	0.1-1 cm	Y	N	52	Died

**Table 2:** Patient characteristics of LGSC compared with HGSC

Clinicopathologic features		LGSC		HGSC		*P* value
Age median(range)		54	(27-77)	56	(36-77)	0.075
FIGO	I	6	23.1%	0	0.0%	0.002
	II	0	0.0%	2	3.8%	
	III	19	73.1%	44	84.6%	
	IV	1	3.8%	6	11.5%	
Family history	Yes	4	15.4%	12	23.1%	0.557
	No	22	84.6%	40	76.9%	
Cytoreduction	R0	13	50.0%	16	30.8%	0.25
	0.1-1 cm	9	34.6%	24	46.2%	
	≥1 cm	4	15.4%	12	23.1%	
Chemosensitivity	Yes	15	57.7%	33	63.4%	0.656
	No	8	30.8%	16	30.8%	
	NA	3	11.5%	3	5.8%	
ER	+	21	80.8%	36	70.6%	0.417
	-	5	19.2%	15	29.4%	
PR	+	9	34.6%	5	9.8%	0.012
	-	17	65.4%	46	90.2%	
AR	+	14	53.8%	17	33.3%	0.089
	-	12	46.2%	34	66.7%	
FSHR	+	21	84.0%	28	54.9%	0.021
	-	4	16.0%	23	45.1%	
LHR	+	17	65.4%	18	35.3%	0.015
	-	9	34.6%	33	64.7%	
GnRHR	+	24	100.0%	40	81.6%	0.026
	-	0	0.0%	9	18.4%	

More LGSC patients presented with an early FIGO stage compared to HGSC patients (*p* = 0.002). The LGSC cases were distributed as follows: stage I, 6 (23.1%); stage II, 0; stage III, 19 (73.1%); and stage IV, 1 (3.8%). The distribution of HGSC FIGO stage was as follows: stage I, 0 (0%); stage II, 2 (3.8%); stage III, 44 (84.6%); and stage IV, 6 (11.5%). There were no significant differences in terms of other basic clinical features including age, family history, surgical outcomes and chemosensitivity between both groups. The median (range) age of LGSC patients was 54 (27–77) years. At the time of surgery, 13 (50%) LGSC patients were debulked to no visible residual disease, and 9 (34.6%) had <1 cm but retained macroscopic disease. With one exception, all LGSC patients received postoperative platinum-based chemotherapy; 15 (57.7%) were platinum sensitive.

**Fig. 1:**
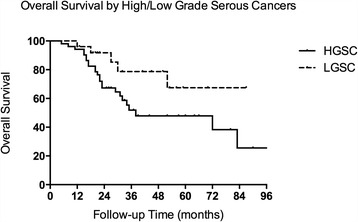
The Kaplan-Meier curve of overall survival for patients with ovarian LGSC and HGSC (*P* = 0.06)

The median (range) follow-up time for LGSC patients was 30 (12–87) months. In the overall cohort, a total of five (19.2%) deaths were documented. The median follow-up time for the matched HGSC cohort was 26 (5–99) months. Twenty-four (46.2%) deaths were documented. The overall survival curves for LGSC and HGSC patients are shown in Fig. 1. The LGSC group presented a longer overall survival rate than the HGSC group, which approached significance (*p* = 0.06). The 2-year and 5-year OSs for LGSC were 91.8% and 67.5%, respectively. The 2-year and 5-year OSs for HGSC were 67.3% and 47.9%, respectively.

We then estimated some potential prognostic clinical factors in LGSC patients. Five out of 20 patients at an advanced FIGO stage (III and IV) died, but no deaths were observed in patients with an early FIGO stage (I and II). Similar results were found for residual disease (R0 *vs* 0.1–1 cm *vs* ≥1 cm: 0/13 *vs* 4/9 *vs* 1/4, *p* = 0.063). Patients with chemosensitive disease had a lower death rate compared to those with chemoresistant disease (6.7% *vs* 37.5%, *p* = 0.117) (Table 3).

**Table 3:** Risk of death categorized by clinicopathologic parameters

Characteristics		Overall death		P (log rank test)
FIGO	I	0/6	0.0%	0.051
	II	0/0	-	
	III	4/19	21.1%	
	IV	1/1	100.0%	
Family history	Yes	0/4	0.0%	0.339
	No	5/22	22.7%	
Cytoreduction	R0	0/13	0.0%	0.063
	0.1-1 cm	4/9	44.4%	
	≥1 cm	1/4	25.0%	
Chemosensitivity	Yes	1/15	6.7%	0.117
	No	3/8	37.5%	
	NA	1/3	33.3%	
ER	+	3/21	14.3%	0.173
	-	2/5	40.0%	
PR	+	0/9	0.0%	0.148
	-	5/17	29.4%	
AR	+	2/14	14.3%	0.508
	-	3/12	25.0%	
FSHR	+	4/21	19.0%	0.862
	-	1/4	25.0%	
LHR	+	3/17	17.6%	0.88
	-	2/9	22.2%	
GnRHR	+	4/24	16.7%	NA
	-	0/0	-	

### Hormone receptor expression

**Fig. 2:**
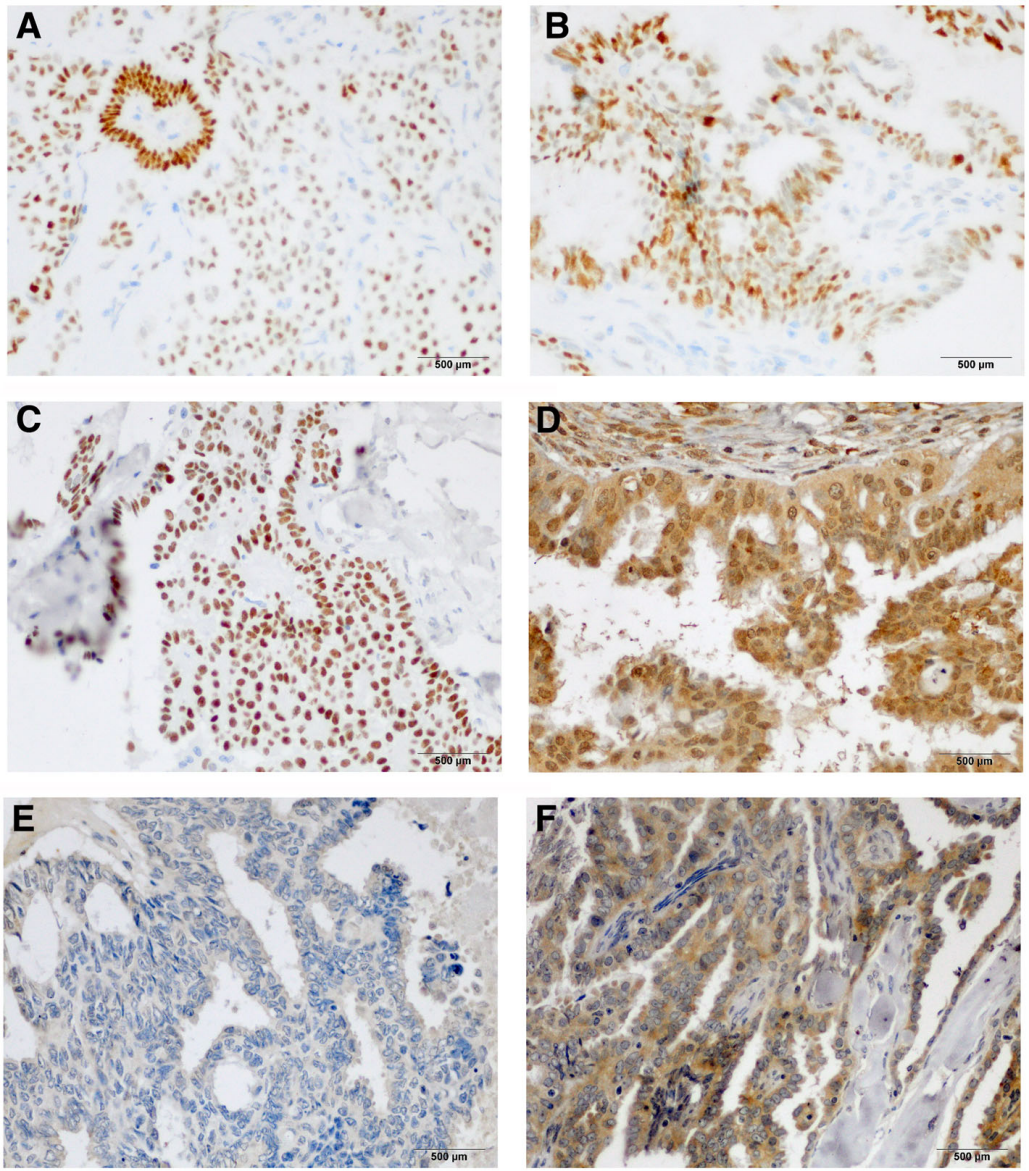
Representative positive staining for ER (**a**), PR (**b**), AR (**c**), FSHR (**d**), LHR (**e**) and GnRHR (**f**) at 400 × magnification (LGSC)

Representative images of LGSC and HGSC hormone receptor staining are shown in Fig. 2 and Additional file 1: Figure S1. The hormonal receptor expression levels of both groups are shown in Table 2 and Additional file 2: Figure S2. A total of 21 (80.8%) LGSC patients were ER positive, compared with 70.6% of HGSC patients (*p* = 0.417). LGSC patients presented much higher expression levels of PR (34.6% *vs* 9.8%, *p* = 0.012), FSHR (84.0% *vs* 54.9%, *p* = 0.021), LHR (65.4% *vs* 35.3%, *p* = 0.015) and GnRHR (100% *vs* 81.6%, *p* = 0.026) compared to HGSC patients. The AR expression level was also higher in LGSC than in HGSC patients, but the difference was not significant (53.8% *vs* 33.3%, *p* = 0.089).

Associations of clinical parameters and hormone receptor expression in LGSC are shown in Additional file 3: Table S1. More patients in the R0 group showed ER positive compared with those who had 0.1–1 cm and ≥1 cm residual disease. There are no other significant differences between hormone receptor expression and clinical parameters.

**Fig. 3:**
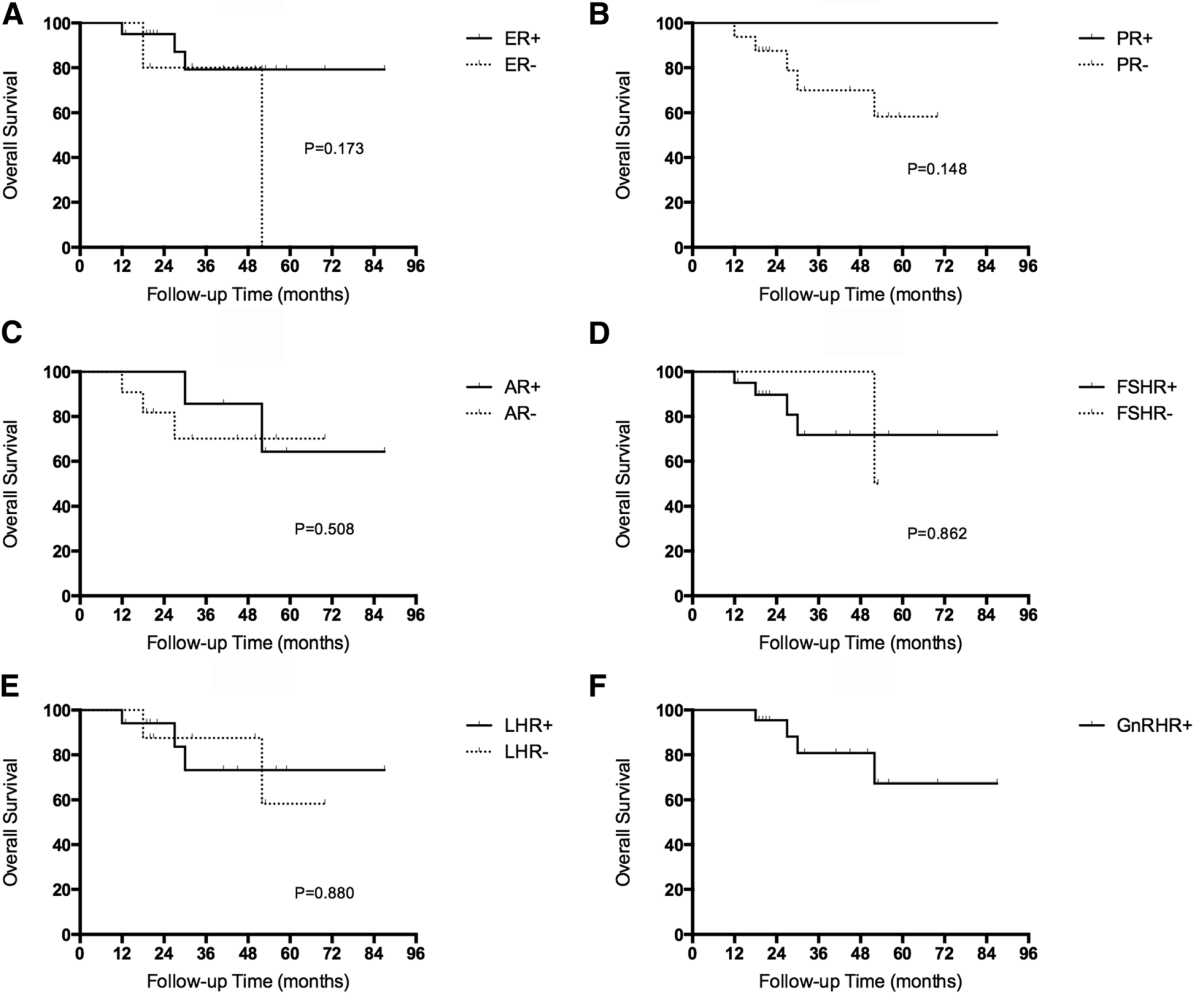
Kaplan-Meier curves of overall survival in LGSC patients stratified by hormone receptor expression (**a** ER, **b** PR, **c** AR, **d** FHSR, **e** LHR, **g** GnRHR)

Data for hormone receptor expression and patient prognosis were also analyzed. The risk of death was higher in hormone receptor negative patients than hormone receptor positive patients: ER- *vs* ER+ 40.0% *vs* 14.3%, PR- *vs* PR+ 29.4% *vs* 0%, AR- *vs* AR+ 25.0% *vs* 14.3%, FSHR- *vs* FSHR+ 25.0% *vs* 19.0%, LHR- *vs* LHR+ 22.2% *vs* 17.6%. However, the differences were not significant in univariate analyses (Fig. 3 and Additional file 3: Table S2). Similarly, there were no significant prognostic factors for HGSC in univariate analyses (Additional file 3: Table S3).

## Discussion

In this mono-institutional study, we investigated the clinicopathologic features of 26 LGSC patients with a higher overall survival rate and distinctive hormone receptor expression levels compared with the HGSC cohort. Six hormone receptors related to the hypothalamic-pituitary-gonadal axis were found to be highly expressed in LGSC. Patients with positive hormone receptor expression tended to have a better prognosis than the corresponding hormone receptor negative patients.

In our cohort, LGSC constituted 2.9% of all serous ovarian cancer, highlighting the rarity of this neoplasm. The incidence rate was quite lower than that reported by other investigators [7, 9, 10, 23]. There was a potential selection bias because we excluded all referral/consultation cases sent to our hospital as well as those who received neoadjuvant chemotherapy. Another explanation may be incidence variation among different ethnicities.

We found that LGSC tended to present with an advanced FIGO stage (FIGO stage III/IV *vs* FIGO stage I: 76.9% *vs* 23.1%), although the rate was lower than HGSC. This finding is in accordance with the distribution obtained in previous studies [23, 24]. Moreover, although LGSC is known to be less sensitive to conventional platinum-based chemotherapy compared to high-grade serous ovarian cancers (HGSC) in the literature, no exact data have been reported [10, 11, 25]. We reported a platinum sensitivity rate of 57.7%, which made the data for the LGSC study more complete. However, our study did not find any significant difference between the two groups. This may be due to the limited number of cases.

The 5-year overall survival rate of LGSC ranges from 61 to 71% in the literature [6, 23, 24, 26]; the 5-year OS observed in our cohort was 67.5%. Similar to previous studies, we confirmed a higher overall survival rate in LGSC compared with HGSC, although this only approached significance [6, 23, 26–28]. In this study, we evaluated the survival in both groups without taking into account FIGO stage, cytoreduction outcomes or other clinicopathologic features in our limited number of study cases.

Furthermore, although several studies have investigated the significance of hormone receptor expression in ovarian cancer, the results are inconsistent, and most studies combined all disease subtypes [17, 29–34]. Studies evaluating the subtype-specific influence on hormone receptor expression are urgently needed. We have previously reported the prognostic significance of hormone receptor expression in HGSC, which is the major subtype of ovarian cancer [35]. As a distinctively different but rare tumor, reports on hormone receptor expression in LGSC are scarce. In our study, LGSC presented significantly greater hormone receptor levels compared to HGSC. We found substantial expression levels of ER (80.8%) and PR (34.6%) in LGSC, which is in accordance with previous studies [13, 14]. In addition, we reported high expression levels of other hormone receptors (AR, FSHR, LHR and GnRHR) related to the hypothalamic-pituitary-gonadal axis for the first time. Interestingly, hormone receptor negative LGSC patients had a higher risk of death compared to hormone receptor positive LGSC patients. Although the survival advantages were not significant, this could indicate the predictive value of these biomarkers. Furthermore, hormone therapy and targeted therapy may be promising alternatives for LGSC treatment. Gershenson et al. [15] reported moderate anti-tumor activity of hormone therapy in patients with recurrent LGSC. FSHR or GnRHR targeted agents have been developed by using corresponding ligands, and phase II studies have shown their promising applications for ovarian cancer [36, 37]. The high expression levels of hormone receptors in our study indicate the potential application of the above treatment in LGSC.

Our study is limited because it is a retrospective study that is dependent on accurate medical records, and thus may have potential recall bias. Although there were only 26 patients in our study, due to the rarity of this tumor, any data related to the clinicopathologic features of LGSC should be reported.

## Conclusions

In conclusion, our study presented a higher overall survival rate and distinctive hormone receptor expression levels of LGSC patients compared with the HGSC cohort. Patients with positive hormone receptor expression tended to have a better prognosis than the corresponding hormone receptor negative patients. Future prospective cooperative multicenter studies are needed to further identify more details about the clinicopathologic characteristics of LGSC.

## Additional files

Additional file 1: Figure S1. Representative positive staining for ER (A), PR (B), AR (C), FSHR (D), LHR (E) and GnRHR (F) at 400x magnification (HGSC). (TIFF 2702 kb)
Additional file 2: Figure S2. Differences of hormone receptor expression levels between LGSC and HGSC groups: ER (A), PR (B), AR (C), FSHR (D), LHR(E) and GnRHR (F). (TIFF 180 kb)
Additional file 3: Table S1. Associations of clinical parameters with hormone receptor expression in LGSC. **Table S2.** Univariate analyses of risk factors for OS in LGSC cases. **Table S3.** Univariate analyses of risk factors for OS in HGSC cases. (DOC 95 kb)

## Abbreviations

AR: Androgen receptor
CI: Confidence interval
ER: Estrogen receptor
FSHR: Follicle-stimulating hormone receptor
GnRHR: Gonadotropin-releasing receptor
HGSC: High-grade serous ovarian cancer
HR: Hazard ratio
IRS: Immunoreactive score
LGSC: Low-grade serous ovarian cancer
LHR: Luteinizing hormone receptor
OS: Overall survival
PFS: Progression-free survival
PR: Progesterone receptor
RD: Residual disease
SI: Staining intensity
