# Supplementary figures and images for: Expression of hypothalamic-pituitary-gonadal axis-related hormone receptors in low-grade serous ovarian cancer (LGSC)
Source: J Ovarian Res. 2017 Jan 25;10:7. doi: 10.1186/s13048-016-0300-5 (PMC5264293; doi:10.1186/s13048-016-0300-5)

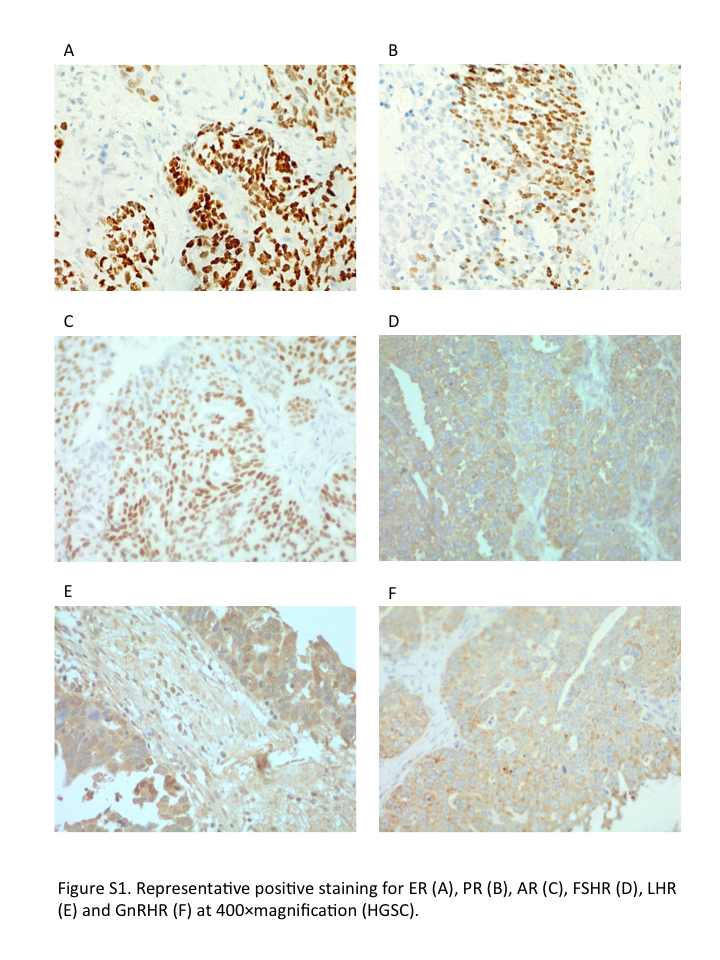

Supplement: Additional file 1: Figure S1. — Representative positive staining for ER (A), PR (B), AR (C), FSHR (D), LHR (E) and GnRHR (F) at 400x magnification (HGSC). (TIFF 2702 kb) [file 13048_2016_300_MOESM1_ESM.tiff]

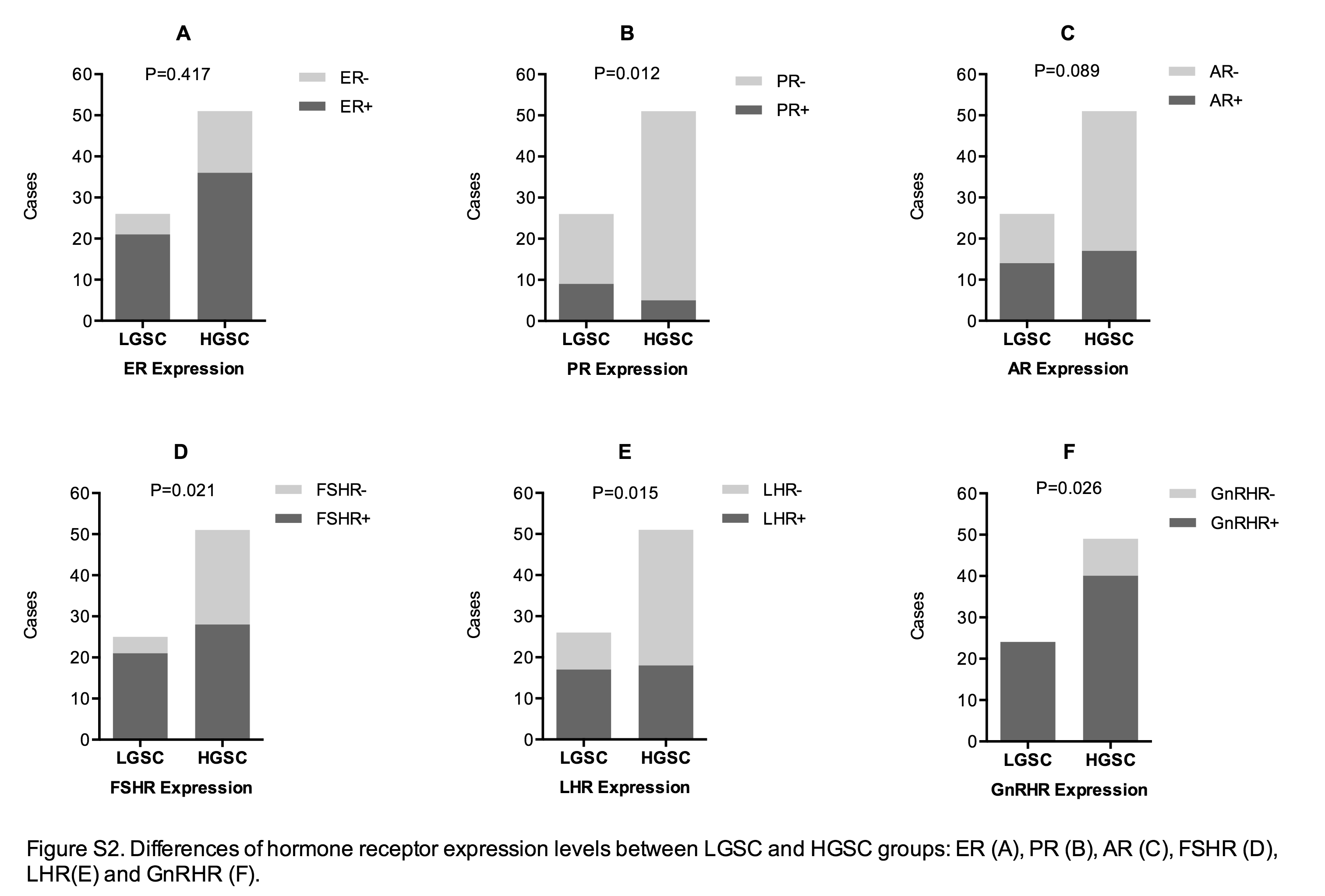

Supplement: Additional file 2: Figure S2. — Differences of hormone receptor expression levels between LGSC and HGSC groups: ER (A), PR (B), AR (C), FSHR (D), LHR(E) and GnRHR (F). (TIFF 180 kb) [file 13048_2016_300_MOESM2_ESM.tiff]
