# Supplementary material for: Expression of hypothalamic-pituitary-gonadal axis-related hormone receptors in low-grade serous ovarian cancer (LGSC)
Source: J Ovarian Res. 2017 Jan 25;10:7. doi: 10.1186/s13048-016-0300-5 (PMC5264293; doi:10.1186/s13048-016-0300-5)
Supplement: Additional file 3: Table S1. — Associations of clinical parameters with hormone receptor expression in LGSC. Table S2. Univariate analyses of risk factors for OS in LGSC cases. Table S3. Univariate analyses of risk factors for OS in HGSC cases. (DOC 95 kb) [file 13048_2016_300_MOESM3_ESM.doc]

| Table S1. Associations of clinical parameters with hormone receptor expression in LGSC | | | | | | | | | | | | | | | | |
| --- | --- | --- | --- | --- | --- | --- | --- | --- | --- | --- | --- | --- | --- | --- | --- | --- |
| Clinical Parameters | | ER | | | PR | | | AR | | | FSHR | | | LHR | | |
|  |  | - | + | P | - | + | P | - | + | P | - | + | P | - | + | P |
| FIGO | I-II | 1 | 5 | 1.000 | 3 | 3 | 0.628 | 2 | 4 | 0.652 | 0 | 5 | 0.549 | 1 | 5 | 0.380 |
|  | III-IV | 4 | 16 |  | 14 | 6 |  | 10 | 10 |  | 4 | 16 |  | 8 | 12 |  |
| Cytoreduction | R0 | 1 | 12 | 0.009 | 6 | 7 | 0.106 | 5 | 8 | 0.721 | 1 | 11 | 0.599 | 4 | 9 | 0.167 |
|  | 0.1-1cm | 1 | 8 |  | 8 | 1 |  | 5 | 4 |  | 2 | 7 |  | 2 | 7 |  |
|  | ≥1cm | 3 | 1 |  | 3 | 1 |  | 2 | 2 |  | 1 | 3 |  | 3 | 1 |  |
| Chemosensitivity | No | 3 | 5 | 0.181 | 7 | 1 | 0.176 | 5 | 3 | 0.221 | 1 | 7 | 1.000 | 4 | 4 | 0.371 |
|  | Yes | 2 | 13 |  | 8 | 7 |  | 5 | 10 |  | 3 | 11 |  | 4 | 11 |  |

| Table S2. Univariate analyses of risk factors for OS in LGSC cases | | | | |
| --- | --- | --- | --- | --- |
| Characteristics | | HR | 95%CI | P |
| FIGO | I | Reference | | |
|  | II | - | | |
|  | III | 56216.348 | (0-7.74E+183) | 0.959 |
|  | IV | 410414.834 | (0-5.68E+184) | 0.951 |
| Family history | Yes | Reference | | |
|  | No | 5.105 | (0.026-986.024) | 0.544 |
| Cytoreduction | R0 | Reference | | |
|  | 0.1-1cm | 215852.457 | (0-1.24E+176) | 0.951 |
|  | ≥1cm | 252594.388 | (0-1.45E+176) | 0.951 |
| Chemosensitivity | Yes | Reference | | |
|  | No | 5.119 | (0.529-49.523) | 0.158 |
| ER | + | Reference | | |
|  | - | 3.259 | (0.539-19.7) | 0.198 |
| PR | + | Reference | | |
|  | - | 36.364 | (0.01-138799.667) | 0.393 |
| AR | + | Reference | | |
|  | - | 1.814 | (0.302-10.883) | 0.515 |
| FSHR | + | Reference | | |
|  | - | 0.822 | (0.090-7.501) | 0.862 |
| LHR | + | Reference | | |
|  | - | 1.148 | (0.191-6.906) | 0.88 |
| GnRHR | + | - | | |
|  | - |  |  |  |

| Table S3. Univariate analyses of risk factors for OS in HGSC cases | | | | |
| --- | --- | --- | --- | --- |
| Characteristics | | HR | 95%CI | P |
| FIGO | I | - | | |
|  | II | Reference | | |
|  | III | 78957.485 | （0-8.81E+174） | 0.955 |
|  | IV | 49563.144 | （0-5.55E+174） | 0.957 |
| Family history | Yes | Reference | | |
|  | No | 2.341 | （0.795-6.896） | 0.123 |
| Cytoreduction | R0 | Reference | | |
|  | 0.1-1cm | 0.567 | （0.217-1.485） | 0.248 |
|  | ≥1cm | 1.137 | （0.41-3.153） | 0.805 |
| Chemosensitivity | Yes | Reference | | |
|  | No | 1.599 | （0.344-7.44） | 0.549 |
| ER | + | Reference | | |
|  | - | 0.461 | （1.57E-01-1.351） | 0.158 |
| PR | + | Reference | | |
|  | - | 24.704 | （1.03E-01-5953.648） | 0.252 |
| AR | + | Reference | | |
|  | - | 1.445 | （5.94E-01-3.519） | 0.417 |
| FSHR | + | Reference | | |
|  | - | 1.058 | （4.72E-01-2.372） | 0.890 |
| LHR | + | Reference | | |
|  | - | 1.239 | （5.26E-01-2.918） | 0.624 |
| GnRHR | + | Reference | | |
|  | - | 0.818 | （2.74E-01-2.447） | 0.720 |
